# Supplementary material for: Distribution and Molecular Identification of Culex pipiens and Culex tritaeniorhynchus as Potential Vectors of Rift Valley Fever Virus in Jazan, Saudi Arabia
Source: Pathogens. 2021 Oct 15;10(10):1334. doi: 10.3390/pathogens10101334 (PMC8540973; doi:10.3390/pathogens10101334)
Supplement: Supplementary file 1 [file pathogens-10-01334-s001.zip › pathogens-1367613-supplementary.pdf]

Supplementary Data

Table S1. Best fit model selection for *C. pipiens* using Maximum Likelihood fits of 24 different nucleotide substitution models.

Model	#Param	BIC	AICc	lnL	*I*	*G*	R	Freq A	Freq T	Freq C	Freq G	A=>T	A=>C	A=>G	T=>A	T=>C	T=>G	C=>A	C=>T	C=>G	G=>A	G=>T	G=>C
T92	57	**2672.2***	2224.9	-1055.3	n/a	n/a	1.50	0.34	0.34	0.16	0.16	0.06	0.03	0.1	0.06	0.1	0.03	0.06	0.22	0.03	0.22	0.06	0.03
T92+G	58	2674.8	2219.6	-1051.6	n/a	0.05	1.56	0.34	0.34	0.16	0.16	0.06	0.03	0.1	0.06	0.1	0.03	0.06	0.22	0.03	0.22	0.06	0.03
T92+I	58	2678.9	2223.7	-1053.7	0.48	n/a	1.52	0.34	0.34	0.16	0.16	0.06	0.03	0.1	0.06	0.1	0.03	0.06	0.22	0.03	0.22	0.06	0.03
HKY	59	2682.7	2219.6	-1050.6	n/a	n/a	1.50	0.29	0.40	0.15	0.16	0.07	0.03	0.1	0.05	0.09	0.03	0.05	0.26	0.03	0.18	0.07	0.03
T92+G+I	59	2683.4	2220.3	-1051.0	0.76	0.59	1.61	0.34	0.34	0.16	0.16	0.06	0.03	0.1	0.06	0.1	0.03	0.06	0.22	0.03	0.22	0.06	0.03
HKY+G	60	2684.8	2213.9	-1046.8	n/a	0.05	1.58	0.29	0.40	0.15	0.16	0.07	0.03	0.11	0.05	0.09	0.03	0.05	0.26	0.03	0.19	0.07	0.03
HKY+I	60	2689.0	2218.1	-1048.8	0.48	n/a	1.52	0.29	0.40	0.15	0.16	0.07	0.03	0.11	0.05	0.09	0.03	0.05	0.26	0.03	0.18	0.07	0.03
TN93	60	2691.1	2220.2	-1049.9	n/a	n/a	1.50	0.29	0.40	0.15	0.16	0.07	0.03	0.14	0.05	0.07	0.03	0.05	0.18	0.03	0.25	0.07	0.03
HKY+G+I	61	2693.2	2214.4	-1046.0	0.77	0.58	1.65	0.29	0.40	0.15	0.16	0.07	0.03	0.11	0.05	0.1	0.03	0.05	0.26	0.03	0.19	0.07	0.03
TN93+G	61	2693.5	2214.7	-1046.2	n/a	0.05	1.56	0.29	0.40	0.15	0.16	0.07	0.03	0.14	0.05	0.07	0.03	0.05	0.19	0.03	0.25	0.07	0.03
TN93+I	61	2697.6	2218.9	-1048.2	0.48	n/a	1.52	0.29	0.40	0.15	0.16	0.07	0.03	0.14	0.05	0.07	0.03	0.05	0.18	0.03	0.25	0.07	0.03
TN93+G+I	62	2702.0	2215.4	-1045.5	0.76	0.58	1.62	0.29	0.40	0.15	0.16	0.07	0.03	0.14	0.05	0.07	0.03	0.05	0.2	0.03	0.25	0.07	0.03
GTR	63	2716.6	2222.2	-1047.9	n/a	n/a	0.95	0.29	0.40	0.15	0.16	0.15	0.02	0.1	0.11	0.07	0.02	0.03	0.2	0.02	0.18	0.06	0.02
GTR+G	64	2719.1	2216.8	-1044.2	n/a	0.05	1.00	0.29	0.40	0.15	0.16	0.15	0.02	0.1	0.11	0.08	0.02	0.03	0.21	0.02	0.18	0.06	0.02
GTR+I	64	2723.1	2220.9	-1046.2	0.48	n/a	0.96	0.29	0.40	0.15	0.16	0.15	0.02	0.1	0.11	0.07	0.02	0.03	0.2	0.02	0.18	0.06	0.02
GTR+G+I	65	2727.1	2217.0	-1043.3	0.75	0.65	1.05	0.29	0.40	0.15	0.16	0.15	0.02	0.1	0.11	0.08	0.02	0.04	0.22	0.02	0.18	0.05	0.02
JC	55	2763.8	2332.1	-1110.9	n/a	n/a	0.5	0.25	0.25	0.25	0.25	0.08	0.08	0.08	0.08	0.08	0.08	0.08	0.08	0.08	0.08	0.08	0.08
K2	56	2765.5	2326.0	-1106.8	n/a	n/a	1.50	0.25	0.25	0.25	0.25	0.05	0.05	0.15	0.05	0.15	0.05	0.05	0.15	0.05	0.15	0.05	0.05
JC+G	56	2766.7	2327.1	-1107.4	n/a	0.05	0.5	0.25	0.25	0.25	0.25	0.08	0.08	0.08	0.08	0.08	0.08	0.08	0.08	0.08	0.08	0.08	0.08
K2+G	57	2769.0	2321.6	-1103.6	n/a	0.05	1.52	0.25	0.25	0.25	0.25	0.05	0.05	0.15	0.05	0.15	0.05	0.05	0.15	0.05	0.15	0.05	0.05
JC+I	56	2769.5	2330.0	-1108.8	0.48	n/a	0.5	0.25	0.25	0.25	0.25	0.08	0.08	0.08	0.08	0.08	0.08	0.08	0.08	0.08	0.08	0.08	0.08
K2+I	57	2771.9	2324.6	-1105.1	0.48	n/a	1.51	0.25	0.25	0.25	0.25	0.05	0.05	0.15	0.05	0.15	0.05	0.05	0.15	0.05	0.15	0.05	0.05

JC+G+I	57	2774.8	2327.5	-1106.6	0.74	0.68	0.5	0.25	0.25	0.25	0.25	0.08	0.08	0.08	0.08	0.08	0.08	0.08	0.08	0.08	0.08	0.08	0.08
K2+G+I	58	2777.1	2321.9	-1102.8	0.74	0.67	1.54	0.25	0.25	0.25	0.25	0.05	0.05	0.15	0.05	0.15	0.05	0.05	0.15	0.05	0.15	0.05	0.05

* Substitution pattern was best depicted at the lowest BIC scores (Bayesian Information Criterion). A discrete Gamma distribution (+G) of 5 rate categories together with assumption of the presence of certain evolutionarily invariables (+I fraction of sites) were used to model the evolutionary rates non-uniformity among sites. This analysis involved 29 nucleotide sequences.

Table S2 Estimates of Evolutionary Divergence between Sequences of *C. pipiens.*

Species 1	Species 2	Evolutionary distance	Geographical location
C. pipiens/2B64-9.20	***C. pipiens/KCH9***	**0.00919443**	**Kenya**
C. pipiens/2B64-9.20	*C. pipiens/NEH12*	0.00919455	Kenya
C. pipiens/2B64-9.20	*C. pipiens/NAH4*	0.00919455	Kenya
C. pipiens/2B64-9.20	*C. pipiens/Port-2168*	0.00919455	Portugal
C. pipiens/2B64-9.20	*C. pipiens/S41*	0.00919455	Turkey
C. pipiens/2B64-9.20	*C. pipiens/NCH10*	0.01073658	Kenya
C. pipiens/2B64-9.20	*C. pipiens/FCH8*	0.01073658	Kenya
C. pipiens/2B64-9.20	*C. pipiens/13-LCO1490-C05-08*	0.01073658	Botswana
C. pipiens/2B64-9.20	*C. pipiens/NIH11*	0.01074280	Kenya
C. pipiens/2B64-9.20	*C. pipiens/Tunisian-isolate*	0.01074286	Tunisia
C. pipiens/2B64-9.20	*C. pipiens/S16*	0.01074299	Turkey
C. pipiens/2B64-9.20	*C. pipiens/S3*	0.01074299	Turkey
C. pipiens/2B64-9.20	*C. pipiens/Isolate-6*	0.01074299	Germany
C. pipiens/2B64-9.20	*C. pipiens/S67*	0.01074299	Turkey
C. pipiens/2B64-9.20	*C. pipiens/S56*	0.01074299	Turkey
C. pipiens/2B64-9.20	*C. pipiens/S43*	0.01074299	Turkey
C. pipiens/2B64-9.20	*C. pipiens/S39*	0.01074299	Turkey
C. pipiens/2B64-9.20	*C. pipiens/S36*	0.01074299	Turkey
C. pipiens/2B64-9.20	*C. pipiens/S13*	0.01074299	Turkey
C. pipiens/2B64-9.20	*C. pipiens/S28*	0.01074299	Turkey
C. pipiens/2B64-9.20	*C. pipiens/wuzhishan*	0.01074299	China
C. pipiens/2B64-9.20	*C. pipiens/S11*	0.01229692	Turkey
C. pipiens/2B64-9.20	*C. pipiens/MI149*	0.01229692	Australia
C. pipiens/2B64-9.20	*C. pipiens/DZB5*	0.01229712	Hungary
C. pipiens/2B64-9.20	*C. pipiens/DZA1*	0.01229712	Hungary
C. pipiens/2B64-9.20	*C. pipiens/S68*	0.01229712	Turkey
C. pipiens/2B64-9.20	*C. pipiens/S38*	0.01229712	Turkey

C. pipiens/2B64-9.20	*C. pipiens/S26*	0.01383200	Turkey

The number of base substitutions per site from between sequences are shown. Analyses were conducted using the Tamura 3-parameter model [24]. This analysis involved 29 nucleotide sequences. Every uncertain position was omitted for each sequence pair using the option of pairwise deletion. Evolutionary analyses were conducted in MEGA X [22].

Table S3. Best fitting model selection for *C. tritaeniorhynchus* using Maximum Likelihood fits of 24 different nucleotide substitution models.

Model	#Param	BIC	AICc	lnL	*I*	*G*	R	Freq A	Freq T	Freq C	Freq G	A=>T	A=>C	A=>G	T=>A	T=>C	T=>G	C=>A	C=>T	C=>G	G=>A	G=>T	G=>C
T92+I	188	3624.7*	2182.3	-900.9	0.45	n/a	1.82	0.34	0.34	0.16	0.16	0.06	0.03	0.11	0.06	0.11	0.03	0.06	0.23	0.03	0.23	0.06	0.03
T92+G	188	3630.9	2188.4	-904.0	n/a	0.56	1.91	0.34	0.34	0.16	0.16	0.05	0.02	0.11	0.05	0.11	0.02	0.05	0.24	0.02	0.24	0.05	0.02
T92+G+I	189	3634.1	2184.0	-900.8	0.42	6.52	1.85	0.34	0.34	0.16	0.16	0.05	0.02	0.11	0.05	0.11	0.02	0.05	0.23	0.02	0.23	0.05	0.02
HKY+I	190	3639.4	2181.6	-898.6	0.45	n/a	1.82	0.37	0.32	0.13	0.18	0.05	0.02	0.12	0.06	0.09	0.03	0.06	0.22	0.03	0.25	0.05	0.02
HKY+G	190	3641.5	2183.8	-899.6	n/a	0.55	1.90	0.37	0.32	0.13	0.18	0.05	0.02	0.13	0.06	0.09	0.03	0.06	0.22	0.03	0.25	0.05	0.02
TN93+I	191	3646.8	2181.4	-897.4	0.45	n/a	1.88	0.37	0.32	0.13	0.18	0.05	0.02	0.15	0.06	0.07	0.03	0.06	0.17	0.03	0.29	0.05	0.02
HKY+G+I	191	3648.8	2183.4	-898.4	0.42	6.53	1.85	0.37	0.32	0.13	0.18	0.05	0.02	0.12	0.06	0.09	0.03	0.06	0.22	0.03	0.25	0.05	0.02
TN93+G	191	3654.8	2189.4	-901.4	n/a	0.56	1.95	0.37	0.32	0.13	0.18	0.05	0.02	0.14	0.06	0.07	0.03	0.06	0.18	0.03	0.29	0.05	0.02
T92	187	3655.8	2221.0	-921.3	n/a	n/a	1.59	0.34	0.34	0.16	0.16	0.06	0.03	0.1	0.06	0.1	0.03	0.06	0.22	0.03	0.22	0.06	0.03
TN93+G+I	192	3656.5	2183.4	-897.4	0.44	14.66	1.90	0.37	0.32	0.13	0.18	0.05	0.02	0.15	0.06	0.07	0.03	0.06	0.17	0.03	0.29	0.05	0.02
K2+I	187	3662.7	2227.9	-924.8	0.46	n/a	1.69	0.25	0.25	0.25	0.25	0.05	0.05	0.16	0.05	0.16	0.05	0.05	0.16	0.05	0.16	0.05	0.05
K2+G	187	3668.6	2233.8	-927.7	n/a	0.59	1.73	0.25	0.25	0.25	0.25	0.05	0.05	0.16	0.05	0.16	0.05	0.05	0.16	0.05	0.16	0.05	0.05
GTR+I	194	3670.5	2182.2	-894.7	0.44	n/a	1.73	0.37	0.32	0.13	0.18	0.04	0.04	0.13	0.04	0.07	0.03	0.11	0.17	0.03	0.27	0.05	0.02
HKY	189	3670.6	2220.5	-919.0	n/a	n/a	1.58	0.37	0.32	0.13	0.18	0.06	0.02	0.12	0.07	0.08	0.03	0.07	0.21	0.03	0.24	0.06	0.02
K2+G+I	188	3670.6	2228.2	-923.9	0.45	39.53	1.69	0.25	0.25	0.25	0.25	0.05	0.05	0.16	0.05	0.16	0.05	0.05	0.16	0.05	0.16	0.05	0.05
GTR+G	194	3672.4	2184.1	-895.7	n/a	0.55	1.82	0.37	0.32	0.13	0.18	0.03	0.04	0.13	0.04	0.08	0.03	0.12	0.19	0.03	0.25	0.05	0.02
GTR+G+I	195	3679.1	2183.1	-894.2	0.42	6.84	1.81	0.37	0.32	0.13	0.18	0.03	0.04	0.14	0.04	0.07	0.03	0.11	0.18	0.03	0.27	0.05	0.02
TN93	190	3681.7	2223.9	-919.7	n/a	n/a	1.58	0.37	0.32	0.13	0.18	0.06	0.02	0.13	0.07	0.08	0.03	0.07	0.19	0.03	0.25	0.06	0.02
K2	186	3690.5	2263.3	-943.5	n/a	n/a	1.53	0.25	0.25	0.25	0.25	0.05	0.05	0.15	0.05	0.15	0.05	0.05	0.15	0.05	0.15	0.05	0.05
JC+I	186	3692.7	2265.6	-944.6	0.45	n/a	0.5	0.25	0.25	0.25	0.25	0.08	0.08	0.08	0.08	0.08	0.08	0.08	0.08	0.08	0.08	0.08	0.08
JC+G	186	3696.8	2269.7	-946.7	n/a	0.64	0.5	0.25	0.25	0.25	0.25	0.08	0.08	0.08	0.08	0.08	0.08	0.08	0.08	0.08	0.08	0.08	0.08
JC+G+I	187	3702.4	2267.6	-944.6	0.45	200	0.5	0.25	0.25	0.25	0.25	0.08	0.08	0.08	0.08	0.08	0.08	0.08	0.08	0.08	0.08	0.08	0.08
GTR	193	3703.2	2222.5	-915.9	n/a	n/a	1.48	0.37	0.32	0.13	0.18	0.05	0.04	0.12	0.05	0.07	0.03	0.12	0.19	0.03	0.23	0.05	0.02

JC	185	3719.4	2299.9	-962.8	n/a	n/a	0.5	0.25	0.25	0.25	0.25	0.08	0.08	0.08	0.08	0.08	0.08	0.08	0.08	0.08	0.08	0.08	0.08

* Models with the lowest BIC scores (Bayesian Information Criterion) are considered to describe the substitution pattern the best. For each model, AICc value (Akaike Information Criterion, corrected), Maximum Likelihood value (lnL), and the number of parameters (including branch lengths) are also presented [23]. Non-uniformity of evolutionary rates among sites may be modeled by using a discrete Gamma distribution (+G) with 5 rate categories and by assuming that a certain fraction of sites are evolutionarily invariable (+I). Whenever applicable, estimates of gamma shape parameter and/or the estimated fraction of invariant sites are shown. They are followed by nucleotide frequencies (f) and rates of base substitutions (r) for each nucleotide pair.Relative values of instantaneous r should be considered when evaluating them. For simplicity, sum of r values is made equal to 1 for each model. For estimating ML values, a tree topology was automatically computed using MEGA X [22]. This analysis involved 67 nucleotide sequences. Codon positions included were 1st+2nd+3rd+Noncoding.

Abbreviations: TR: General Time Reversible; HKY: Hasegawa-Kishino-Yano; TN93: Tamura-Nei; T92: Tamura 3-parameter; K2: Kimura 2-parameter; JC: Jukes-Cantor

Table S4. Estimates of Evolutionary Divergence between Sequences of *C. tritaeniorhynchus*.

Species 1	Species 2	Evolutionary distance	Geographical location
C. tritaeniorhynchus/2B64-9.20	***C. tritaeniorhynchus/MBIM1-A3***	**0.00464026**	**Turkey**
C. tritaeniorhynchus/2B64-9.20	*C. tritaeniorhynchus/MBIM1-A7*	0.00776174	Turkey
C. tritaeniorhynchus/2B64-9.20	*C. tritaeniorhynchus/MBIM1-A5*	0.00933086	Turkey
C. tritaeniorhynchus/2B64-9.20	*C. tritaeniorhynchus/SYYK-5*	0.00933140	China
C. tritaeniorhynchus/2B64-9.20	*C. tritaeniorhynchus/YZFS-14*	0.01088612	China
C. tritaeniorhynchus/2B64-9.20	*C. tritaeniorhynchus/SXAK-2*	0.01090597	China
C. tritaeniorhynchus/2B64-9.20	*C. tritaeniorhynchus/BJMJ-5*	0.01090597	China
C. tritaeniorhynchus/2B64-9.20	*C. tritaeniorhynchus/544IRI2011*	0.01090634	Japan
C. tritaeniorhynchus/2B64-9.20	*C. tritaeniorhynchus/YZFS-5*	0.01090634	China
C. tritaeniorhynchus/2B64-9.20	*C. tritaeniorhynchus/SCYA-16*	0.01090634	China
C. tritaeniorhynchus/2B64-9.20	*C. tritaeniorhynchus/SCNC-11*	0.01090634	China
C. tritaeniorhynchus/2B64-9.20	*C. tritaeniorhynchus/NX-9*	0.01090634	China
C. tritaeniorhynchus/2B64-9.20	*C. tritaeniorhynchus/LHWY-4*	0.01090634	China
C. tritaeniorhynchus/2B64-9.20	*C. tritaeniorhynchus/LHLW-1*	0.01090634	China
C. tritaeniorhynchus/2B64-9.20	*C. tritaeniorhynchus/GZYC-23*	0.01090634	China
C. tritaeniorhynchus/2B64-9.20	*C. tritaeniorhynchus/GXBH-18*	0.01090634	China
C. tritaeniorhynchus/2B64-9.20	*C. tritaeniorhynchus/GXBH-17*	0.01090634	China
C. tritaeniorhynchus/2B64-9.20	*C. tritaeniorhynchus/GSPL-16*	0.01090634	China
C. tritaeniorhynchus/2B64-9.20	*C. tritaeniorhynchus/FJND-8*	0.01090634	China
C. tritaeniorhynchus/2B64-9.20	*C. tritaeniorhynchus/BJMJ-3*	0.01090634	China
C. tritaeniorhynchus/2B64-9.20	*C. tritaeniorhynchus/ZJNB-5*	0.01090672	China
C. tritaeniorhynchus/2B64-9.20	*C. tritaeniorhynchus/SYPJ-19*	0.01090672	China
C. tritaeniorhynchus/2B64-9.20	*C. tritaeniorhynchus/ZJNB-3*	0.01248697	China
C. tritaeniorhynchus/2B64-9.20	*C. tritaeniorhynchus/ZJJH-4*	0.01248697	China
C. tritaeniorhynchus/2B64-9.20	*C. tritaeniorhynchus/ZJJH-2*	0.01248697	China
C. tritaeniorhynchus/2B64-9.20	*C. tritaeniorhynchus/ZJJH-23*	0.01248697	China
C. tritaeniorhynchus/2B64-9.20	*C. tritaeniorhynchus/ZJJH-13*	0.01248697	China
C. tritaeniorhynchus/2B64-9.20	*C. tritaeniorhynchus/YZFS-9*	0.01248697	China
C. tritaeniorhynchus/2B64-9.20	*C. tritaeniorhynchus/YZFS-3*	0.01248697	China
C. tritaeniorhynchus/2B64-9.20	*C. tritaeniorhynchus/YNRL-21*	0.01248697	China
C. tritaeniorhynchus/2B64-9.20	*C. tritaeniorhynchus/YNRL-15*	0.01248697	China
C. tritaeniorhynchus/2B64-9.20	*C. tritaeniorhynchus/SYYK-1*	0.01248697	China
C. tritaeniorhynchus/2B64-9.20	*C. tritaeniorhynchus/SYYK-11*	0.01248697	China
C. tritaeniorhynchus/2B64-9.20	*C. tritaeniorhynchus/SYPJ-8*	0.01248697	China
C. tritaeniorhynchus/2B64-9.20	*C. tritaeniorhynchus/SYPJ-3*	0.01248697	China
C. tritaeniorhynchus/2B64-9.20	*C. tritaeniorhynchus/SYPJ-22*	0.01248697	China
C. tritaeniorhynchus/2B64-9.20	*C. tritaeniorhynchus/SYPJ-13*	0.01248697	China

C. tritaeniorhynchus/2B64-9.20	*C. tritaeniorhynchus/SYJZ-7*	0.01248697	China
C. tritaeniorhynchus/2B64-9.20	*C. tritaeniorhynchus/SYJZ-2*	0.01248697	China
C. tritaeniorhynchus/2B64-9.20	*C. tritaeniorhynchus/SYJZ-19*	0.01248697	China
C. tritaeniorhynchus/2B64-9.20	*C. tritaeniorhynchus/SYDD-8*	0.01248697	China
C. tritaeniorhynchus/2B64-9.20	*C. tritaeniorhynchus/SXAK-13*	0.01248697	China
C. tritaeniorhynchus/2B64-9.20	*C. tritaeniorhynchus/SXAK-6*	0.01248697	China
C. tritaeniorhynchus/2B64-9.20	*C. tritaeniorhynchus/SHJD-4*	0.01248697	China
C. tritaeniorhynchus/2B64-9.20	*C. tritaeniorhynchus/SHBS-20*	0.01248697	China
C. tritaeniorhynchus/2B64-9.20	*C. tritaeniorhynchus/SCYA-13*	0.01248697	China
C. tritaeniorhynchus/2B64-9.20	*C. tritaeniorhynchus/SCYA-6*	0.01248697	China
C. tritaeniorhynchus/2B64-9.20	*C. tritaeniorhynchus/SCYA-3*	0.01248697	China
C. tritaeniorhynchus/2B64-9.20	*C. tritaeniorhynchus/SCYA-1*	0.01248697	China
C. tritaeniorhynchus/2B64-9.20	*C. tritaeniorhynchus/SCCD-30*	0.01248697	China
C. tritaeniorhynchus/2B64-9.20	*C. tritaeniorhynchus/SCCD-31*	0.01248697	China
C. tritaeniorhynchus/2B64-9.20	*C. tritaeniorhynchus/NX-8*	0.01248697	China
C. tritaeniorhynchus/2B64-9.20	*C. tritaeniorhynchus/NX-7*	0.01248697	China
C. tritaeniorhynchus/2B64-9.20	*C. tritaeniorhynchus/NX-5*	0.01248697	China
C. tritaeniorhynchus/2B64-9.20	*C. tritaeniorhynchus/LHLW-16*	0.01248697	China
C. tritaeniorhynchus/2B64-9.20	*C. tritaeniorhynchus/LHLW-14*	0.01248697	China
C. tritaeniorhynchus/2B64-9.20	*C. tritaeniorhynchus/LHLW-4*	0.01248697	China
C. tritaeniorhynchus/2B64-9.20	*C. tritaeniorhynchus/LHJS-14*	0.01248697	China
C. tritaeniorhynchus/2B64-9.20	*C. tritaeniorhynchus/LHJS-12*	0.01248697	China
C. tritaeniorhynchus/2B64-9.20	*C. tritaeniorhynchus/JXNC-17*	0.01248697	China
C. tritaeniorhynchus/2B64-9.20	*C. tritaeniorhynchus/JSWX-25*	0.01248697	China
C. tritaeniorhynchus/2B64-9.20	*C. tritaeniorhynchus/JSWX-23*	0.01248697	China
C. tritaeniorhynchus/2B64-9.20	*C. tritaeniorhynchus/HNCS-41*	0.01248697	China
C. tritaeniorhynchus/2B64-9.20	*C. tritaeniorhynchus/HNCS-33*	0.01248697	China
C. tritaeniorhynchus/2B64-9.20	*C. tritaeniorhynchus/HNCS-28*	0.01248697	China
C. tritaeniorhynchus/2B64-9.20	*C. tritaeniorhynchus/HNCS-25*	0.01248697	China

The number of base substitutions per site from between sequences are shown. Analyses were conducted using the Tamura 3-parameter model [24]. This analysis involved 67 nucleotide sequences. Codon positions included were 1st+2nd+3rd+Noncoding. All ambiguous positions were removed for each sequence pair (pairwise deletion option). Evolutionary analyses were conducted in MEGA X [22].
